# The Frantic Seeking of Credit during Poker Machine Problem Gambling: A Public Health Perspective

**DOI:** 10.3390/ijerph17145216

**Published:** 2020-07-19

**Authors:** Jane Oakes, Rene Pols, Sharon Lawn

**Affiliations:** 1 PsychMed, Wellbeing and Recovery Research Institute—WARRI, Adelaide, SA 5000, Australia; 2 Department of Psychiatry, College of Medicine and Public Health, Flinders University, P.O. Box 2100, Adelaide, SA 5001, Australia; rgpols@tpg.com.au; 3 College of Medicine and Public Health, Behavioural Health, Flinders University, P.O. Box 2100, Adelaide, SA 5001, Australia; sharon.lawn@flinders.edu.au

**Keywords:** addiction, finances, gambling, money, automated teller machines (ATMs), harms

## Abstract

(1) Background: Financial harms associated with problem gambling are substantial and result in suicidal ideation, depression, anxiety and relationship damage, causing distress for problem gamblers and their families. This paper examines Electronic Gaming Machine gamblers’ frantic use of credit during episodes of gambling as a substantial public health burden. (2) Methods: This qualitative study comprised 29 participants purposefully selected who participated in either focus groups or in-depth interviews, which were analysed using thematic, textual analysis. (3) Results: Ready access to credit in the gambling venues enabled problem gamblers to engage in desperate credit transactions to continue to gamble. Many showed frantic, repeated patterns of e-credit withdrawal, which may be typical of gambling while “in the zone”, when it is highly likely that the gamblers are not able to make informed decisions about the use of credit. This pattern of the electronic withdrawal of cash may well be recognisable electronically by financial institutions in real-time, as part of a duty of care potentially owed by banks to their customers. It would provide an opportunity for the identification of people at financial risk due to gambling and systemic intervention to limit the financial harm at a time when financial decision-making is impaired. (4) Conclusions: Although this finding needs further confirmation, there are significant implications for harm minimisation and early intervention for affected PGs. It also raises the issue of the ‘duty of care’ owed to PG customers by financial institutions.

## 1. Introduction

In Australia, it is estimated that 1.1 million regular gamblers have engaged in gambling where they are at risk of gambling-related problems. This paper examines, how gamblers are significantly impacted by easy access to credit, particularly access within gambling venues. For example, the financial impact of gambling on gamblers and their households is profound. In Australia, problem gamblers (PGs) spend more than four times on gambling compared to those without problems, spending an average of 27% of their disposable household income on gambling; an amount comparable to four times their annual household utility costs (e.g., electricity, gas), or more than half their grocery costs, often defaulting on these essential expenditures [1]. This issue is not a new finding, as financial counsellors have been working with PGs since 1995 [2]. The Australian Productivity Commission [3] established that 70% of PGs were spending more than was affordable, creating many problems for themselves and approximately seven others in their social networks. In a more recent study, the burden of harm from gambling to PGs was similar to major depressive disorders and alcohol use and dependency and included relationship damage, distress, health, and financial impacts [4,5]. This financial stress is associated with much harm to mental health and wellbeing including suicidal ideation, depression, and anxiety [4,6]. An important scoping study has brought much of the evidence together about PGs’ chronic credit misuse, highlighting the responsibility that financial institutions have towards these vulnerable customers and as a significant issue for consumer advocacy groups [7].

The knowledge that EGM PGs are serious misusers of credit has existed for more than 25 years [2,3,8], but little follow-through has occurred other than the piecemeal provision of financial counselling. In the Problem Gambling Financial Counselling Survey and Case Studies report [8], investigating Australian experiences of gambling and financial stress, counsellors reported high use of credit by PGs, with 90% presenting to counselling services because of an inability to pay routine bills. To support their gambling, many PGs borrowed money from commercial sources, including credit cards, payday loans, personal loans, or informal loans from significant others. Client debts ranged from USD 500 for utility debts to a secured loan default of hundreds of thousands of dollars [8].

Financial counselling can reduce these harms from problem gambling [8,9]. However, the Australian Government provides minimal funding for specialised problem gambling financial counsellors who provide information, support and advocacy to help people deal with their financial situations and minimise the risk of future financial problems. This is despite this intervention improving the financial position of 87% of PGs receiving specialised support, and 30% reducing their gambling activity [9]. There have not been any systemic, regulatory approaches towards the banking industry to reduce gambling harm. The chronic, insidious, increasingly unwise, secured and unsecured lending, and accumulation of debt is the typical chronic pattern of credit use by PGs, and it has documented for a long time [7].

Treatment-seeking by PGs is paradoxically extremely low [10] and delayed for up to 5 years [11]. The liberal provision of credit to PGs delays the recognition of the problem, and thereby financial institutions unwittingly prolong problem gambling by consumers. The stigma associated with problem gambling also poses a barrier to disclosure and again delays PGs’ treatment-seeking and recovery [12]. Eventually, professional help-seeking is usually driven by crisis, rather than by the PG’s recognition of problematic behaviour [13]. This lack of insight and self-scrutiny is serious, and compounds problems over time. Early interventions when problematic credit use occurs could lead to the earlier recognition of problem gambling and better outcomes.

Swanton et al. [7] highlighted the need for qualitative methodologies, such as interviews and focus groups, to gain a better understanding of the underlying issues concerning both financial institutions and gambling consumers. Early indicators of problem gambling are patterns of risky credit practices. These are of two types: the first is chronic, insidious, increasingly unwise credit use, as described above; the second type described in this paper involves the frantic seeking of credit during gambling episodes.

Schüll [14] used extensive ethnographic and participant observational work where relationships were developed with many EGM PGs over a considerable period. She described a pattern of credit use confirmed in this study comprising the frantic and atypical accessing of credit during an acute gambling episode when the PG was in what she concluded to be “the machine zone”. Episodes of such credit use were documented in the consumer’s bank statements [14] (p. 71). The PGs repeatedly accessed an Automated Teller Machine (ATM) to withdraw small sums (USD 20–50, an atypical pattern), again and again until all available credit and money were lost, ending the gambling episode. Building on this work [14], the current study also documents this frantic credit-seeking and endorses the review by Swanton et al., which highlighted the duty of care of credit providers [7].

The aim of this study was to gain some understanding of this consumer behaviour when PGs are gambling and are in “the zone”. It is proposed that when in “the zone”, it is highly likely that PGs cannot make informed decisions about the use of credit. This atypical pattern of credit use may be pathognomonic of EGM PGs while they are playing, and there are potential financial interventions that could act to reduce harm for PGs.

## 2. Materials and Methods

The current paper draws on findings from the larger qualitative dataset collected as part of an Australian study on gambling relapse [15,16,17,18,19], using a grounded theory design, that provides participants’ accounts of their use of ATMs and credit as part of their gambling. The use of a qualitative methodology enabled the researchers to listen to and interpret what participants had said about their experiences of EGM problem gambling [20]. The first phase of this study used focus groups to obtain an initial description of the relapse processes. The second phase employed in-depth interviews to provide a further, deeper understanding of relapse, as the researcher and participants engaged in a dialogue in which the researcher could probe interesting and important areas that had been discovered in the focus group component of the study. Data from the in-depth interviews strengthened the focus group findings with rich data about the direct experiences of participants with regards to EGM problem gambling [21].

Decisions about recruitment method and sample size were based on a qualitative methodology, which uses small sample sizes selected purposefully to permit enquiry into, and to understand, a phenomenon in great depth. Purposive sample selection [22] was therefore used for this study to examine relapse from multiple perspectives, and to ascertain how the process of relapse occurs.

This study involved the views and experiences of 29 PGs who partook in either one of three focus groups (n = 10) (Table 1) or an in-depth interview (n = 19) (Table 2). The PGs included relapsed treatment seekers (TS) who had decided to stop gambling and had sought treatment, counselling or self-help programs (Pokies Anonymous (PA), as well as non-treatment seekers (NTS). The larger project’s methods are described in more detail elsewhere [18,19].

All interviews were audio-recorded, with participants’ consent. A professional transcription service was used to transcribe the audio-files of the recorded interviews and focus group discussions. All data were analysed using thematic analysis. Coding of the transcribed data was then undertaken manually by the first two authors who each read and independently open coded all focus group data and then met to resolve any discrepancies prior to further coding levels. The three authors independently open-coded the first six interview transcripts, meeting regularly to discuss and debate the coding process, reaching agreement on tentative codes that the first author then applied to further interview transcripts. Through this process of open coding, axial coding, and constant comparative analysis, systematic generation of preliminary theories from the data occurred that involved both inductive and deductive thinking, and overall synthesis of the data [23]. The comparison of categories between focus groups was undertaken as part of the larger study from which this data on credit use has been drawn. However, reporting on those comparisons was not the subject of this specific study and is therefore not reported in detail here.

The process of comparative reflection on the transcribed focus group and interview data against each other, the literature, and from the ongoing discussions between the researchers, continued until no further new data or themes emerged [24]. Through a process of back and forward discussion as they undertook the cyclic analysis of focus group and then interview data, the research team determined that data saturation was reached once 15 interviews had been conducted. Four further interviews were conducted to ensure the strength of this decision to cease further recruitment to interviews.

Triangulation of the data ensured the results were rich, robust, comprehensive, and well-developed. The data were verified from several sources including the focus group participants, the participants from the in-depth interviews, by various sources which included consultations with the research team and an extensive literature review encompassing ideas from several different paradigms [25]. An external auditor reviewed the fidelity of the methodology and analytic process, confirming that a clear audit trail had been established [25]. 

## 3. Results

The following section uses participants’ quotes to outline PGs’ specific experiences concerning the use of ATMs and e-credit that enabled them to finance gambling despite the financial harms. Data directly relevant to credit use are captured across three main themes. The first theme describes the frantic accessing of credit during the process of gambling. The second theme describes PGs’ reactions once away from the gambling venue when they realise what has occurred from viewing the bank statement evidence. The third theme captures the PGs’ sense of what has occurred in these two processes.

### 3.1. Frantic Accessing of Credit While Gambling

Many participants described a pattern of e-credit use from ATMs, Electronic Funds Transfer at Point of Sale (EFTPOS) facilities, credit or other bank accounts that involved the repeated withdrawal of small amounts of cash until access to credit was no longer available. John (TS: I, aged 42) desperately chased his losses:

*I wasn’t aware of my surroundings or anything, just back and forth to the ATM—$50, 50, 50, 50, 50—desperate in the end. Yeah, that ugly ‘zone’; a dangerous place to be. The ‘zone’ is when you lose rational thinking, and you don’t have the power or the thought process to be able to leave.*

Lee (PA: I, aged 60) emptied his bank account:

*Well, what would happen is that you’d play all your money, empty your bank book, empty that out because they’ve got the ATMs virtually next door to you. Then you try to chase it up, and then I keep trying to chase and chase and chase it up, going back and forth to the auto bank.*

*He also routinely used credit advances:*

*I’d blown $300, or two or $300, from the credit card …..I said to myself ‘look if I ever go back to the ATM and withdraw from the credit card then I’m going to have to go and see ‘somebody’’ so I sort of – and that happened for quite some time.*

Lee described his disbelief at his pattern of withdrawing money from the ATM:

*Oh my God, I’m taking more money out. I’ve gone back to the ATM three times in a night, so at each point, there’s a level of – did pride come into it? I don’t know; I’m trying to recall the feelings. Towards the end – I suppose scared is not quite the right word. I can’t think of how I’d feel at the ATM.*

Irene (NTS: I, aged 65) described her ATM use as a behaviour that was beyond her control:

*I just kept losing. I kept going to the ATM – and, worse, I kept going to the ATM, getting more money out and going back and spending it.*

Monica (PA: I, aged 36), described a similar sense of being detached from her cash withdrawal behaviour at the time:

*Yeah, more than I had. I’d bleed the bank dry. Not good; not good but then I’d think ‘oh okay, a few dollars or $50 I might win some of that back’ so I guess at the time you – yeah I was – I mean I didn’t feel good because I’d be watching the bank account go down and down and down.*

Larry (PA: FG, aged 65) described the dangers of EFTPOS facilities in the hotel to continue gambling at a time when all he could see was the prospect of winning:

*I, you put your money through and think ‘oh I need some more money’ so you go and grab some more money, you use your EFTPOS card and you go and grab some more money, and then you put more money in. You’re going ‘these free games have got to come up’ so you go and get more money and put it in the machine… sometimes I’ve gone overboard, and I might have had to get a credit advance or something like that on a Bankcard.*

### 3.2. Financial Statements Tell the Gambler What They Have Done

When participants eventually received a bank statement, they described being overwhelmed. Colleen (NTS: I, aged 51) was in disbelief at how much she had spent and lost:

*I got a bank statement which showed me what I’d earned and what I still had in the account, and it was like ‘oh my God, I can’t possibly have earned that amount of money and spent that amount of money and I did’.*

Wally (TS: I, aged 40) was shocked, only realising the extent of his losses when he saw his bank statement:

*I had that bank statement sitting in front of me going ‘oh my God, you did lose that amount of money’.*

Brenda (PA: I aged 57) remembered how upset she was to see a receipt showing she had no money left. She saw on the statement how she thought she was ‘in control’ at the time, but now saw how that was simply not the case:

*I could have my bank statements now you’d see that I was going back and forth taking out $20, not wanting to spend the whole lot but eventually spending the whole lot. When the receipt came out, there was no money left; it felt so bad.*

### 3.3. Inability to Make Rational Financial Decisions

Natasha (PA: I, aged 46) questioned her cognitive processes:

*I would say ‘quick I need more money’. I couldn’t get it into the machine quick enough but now to lose that particular amount of money in one day to me, I can’t even understand how my cognitive processes were going at that time. It seems insane.*

When questioned about her awareness, Irene (NTS: I, aged 65) advised:

*No; no, until afterwards, until I realised all that money’s gone. Shocking isn’t it? That was all I had basically, so I had to stop.*

The data provided by participants suggest two key issues regarding e-credit use by PGs.

## 4. Discussion

### 4.1. The Frantic Use of Credit during the Gambling Episode

The thought of money being available, or money becoming ‘available’ in fantasy, allowed the PG to regain their emotional equilibrium at the end of a gambling episode because of an underlying belief that there was a mythical win waiting for them the next time they gambled. They believed that that this would make everything all right again. This enabled the PG to defer playing, and with it the urge to gamble, so the PG could engage in the process of planning the next gambling episode free from the urge to gamble because they had to focus on converting a sum of fantasied “available money” into realistically “accessible money”. The fantasy of “available money” was a critical facilitatory cognition that, together with the underlying belief that there was a win waiting for them to access through gambling, enabled the suspension of the urge to gamble. This hypothetical money was not actually available but was potentially so. It is money needing to be cobbled together through deferral of bills, borrowing, stealing, or waiting for payment of salary or benefits, or some other way. From the moment that “available money” actually became “accessible”, the deferred urge to gamble returned with full force, leading to a vicious cycle of gambling once money was accessed [16]. Access to credit enabled them to engage in desperate credit transactions to continue to gamble. Schüll [14] initially described this pattern of ATM use. Many of our subjects repeatedly withdrew small amounts of money from the ATM, bank account, debit or EFTPOS facility located near a gambling venue while they were actively gambling and chasing losses. This study replicated the work of Schüll [14] using a different methodology and occurring in a different country. The pattern, location (near a gambling venue), and time (often shortly after funds become accessible at midnight), may well be pathognomonic of an acute gambling episode or a specific indicator that the client has a problem with EGM gambling.

### 4.2. Model of Financial Destruction

**Figure 1:**
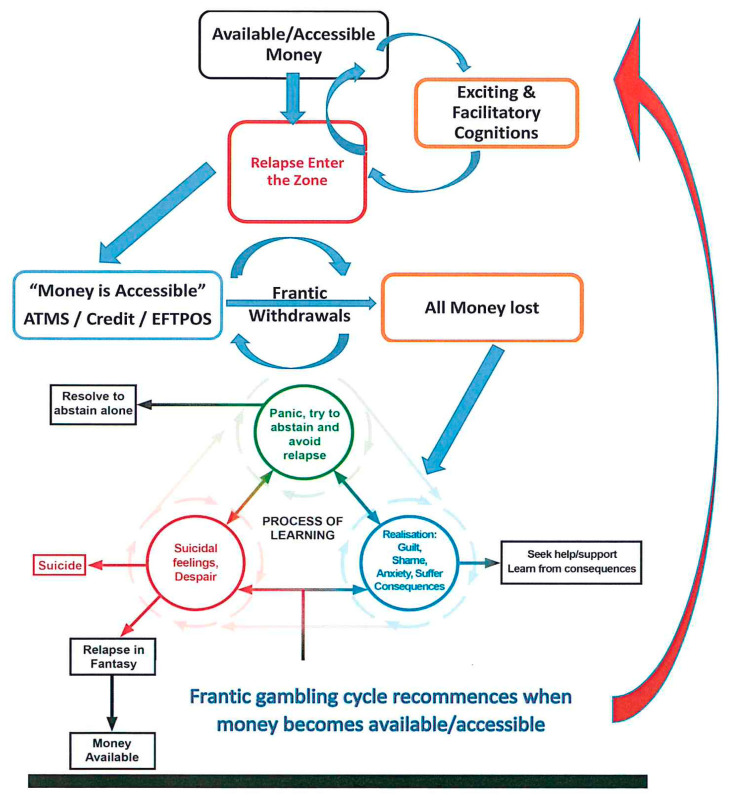
Financial Destruction and Problem Gamblers: A Model.

This model provides new knowledge about how a gambler can continue to use all available money and then credit to continue to gamble despite the harms. This diagram builds on the “merry-go-round” of relapse [15]. This is a complex interplay of mutually reinforcing, and conflicting cognitions that are permission-giving and justifying, allowing PGs to create available money to gamble when this is not the case [16]. The interaction starts when the PG finally receives actual money (for example, when they receive pay from their employment), and is maintained by the ability to access credit. Figure 1 shows how “available money” is the critical factor reinforcing the relapse cycle leading to financial devastation.

When the PG has access to money, they immediately enter the zone [17], an altered state of consciousness where informed decisions about the use of credit are not possible. The gambler then makes repeated and frantic withdrawals of cash until they exhaust all accessible money and, in desperation, uses all accessible credit in an attempt to win back losses, in the desperate belief that the win that will make everything all right again is just around the corner, perhaps on the next spin! When money is no longer accessible, the gambler focuses on money in fantasy until they can source actual money. The process of repeated relapse provides the gambler with an escape from experiencing despair. At this time, self-observation is not possible, and the PG is unable to learn from the damages as a result of their gambling. However, for some, the realisation of harms can slowly begin as they resolve to abstain. However, easy access to money maintains the cycle and prevents the gambler from the self-reflection (Figure 1 Blue Circle: Realisation) necessary to break the gambling cycle.

Once access to money is not possible, a return of critical thinking can result. The PG is then faced with the realisation of the consequences of their behaviour. This realisation and insight creates a significant negative affective state, which is extremely distressing for the PG as they acutely experienced considerable guilt, grief, and shame associated with their relapse. For the PG to move into recovery, hope had to outweigh the belief in the “mythical win”, or this belief had to be suspended or deferred, and this seemed much more possible with support.

Some PGs experienced severe anxiety (Figure 1 Green Circle: Panic) about how they would deal with their desperate financial state which, for most, was intolerable. As the PG realised the extent of what they had done yet again, their distress often escalated, resulting in suicidal feelings to escape from this desperate situation (Figure 1 Red Circle). The choices at such times appeared limited: one where the PG tried to remain abstinent out of shock or fear; a second, where the PG gives in to the despair in her/his life and decides to attempt suicide; the third and most common choice is that the PG tries to heal this despair by starting their next relapse almost immediately after the last has been completed by fantasying about the next episode when money becomes available, and the win could come true. A final option that appears to take many PGs a long time to learn is to stop the process of relapse by facing their despair, responsibility, and loss by seeking help and support to assist them in exiting this desperate way of living. To do this, the capacity to maintain the critical thinking involved in learning is required. If the PG can learn to tolerate their distress, they could then begin to acknowledge the impact of habitual relapse (Blue Circle: Realisation). At this time, they could engage in self-observation where a process of learning from the harm (Figure 1 Centre) caused by their behaviour begins. At this time, the PG tries to remain abstinent and may start to actively engage in the change process towards recovery. Learning to experience emotional states and engage in self-observation did not happen all at once, but was built upon and shaped by the PG’s motivation to continue to be vigilant to the risks of available money rather than escaping back to relapse in fantasy until the opportunity to relapse occurred in reality. It is unclear how the cycle of insight and realisation can be enhanced. The natural history of EGM problem gambling suggests that this is a process that takes some years of repeated losses that can enable learning to occur [15]. The critical role of support [15] allows PGs to experience the negative consequences of their gambling behaviour “in sizeable bits”, so that they slowly experience shame and guilt, rather than fantasising about the next gambling episode and creating “available money” in their wish-fulfilling fantasies. This requires support and acceptance or forgiveness as a way forward, by any therapist and, most importantly, significant others.

### 4.3. Do Financial Institutions Have a Duty of Care to Intervene?

This pattern of PGs accessing credit may be able to be identified electronically in real-time by the financial institution. If so, then a strong case could be made that those institutions should be aware that such patterns of credit use are indicative of PG behaviour by the bank customer. As indicated above, a systemic, electronic intervention could be developed to intercept the out-of-control e-credit use that the PG continues to inflict upon their financial situation and family. Suspending access to credit, and the use of an electronic card which requires a face-to-face review of their credit status could be required for such customers to be able to reinstate their access to electronic fund withdrawal once they can establish the safe use of those funds. However, such a move would require significant consultation and consideration, given the civil liberties implications for bank customers more broadly. At such a time, referral for financial counselling and professional treatment could occur. Previous work by our group has shown that, during relapse there are several cognitions within the lexicon of EGM PGs that distort reality when they are gambling: ‘money’ has a different meaning (it is not ‘real money’ somehow), and that ‘winning’ means winning ‘that will make everything all right again’, undoing all the harm that has accumulated [16].

Interestingly, Schüll [14] (p. 71) described a bank statement as a chronological index of a PG’s withdrawals while in “the zone”, where one of her study participant’s spikes of urgency that drove her trips to the ATM and the periods of suspension between them were recorded. Such banking records may well go back many years and require the “making good” of substantial sums should any class actions by PGs be successful in demonstrating a lack of financial capacity when PGs are in “the zone” while gambling.

This study has not reported the general use of credit by subjects, as this insidious behaviour has been well established over a long time. This knowledge of harmful credit practices by PGs goes back at least to 1995 [2] and to the Australian Gambling Productivity Commission report [3]. The recent international scoping review [7] and clinical reports [9] have highlighted this issue again, and more recently it was highlighted that debt problems are potential precipitants and a consequence of problem gambling, and consumer credit products exacerbate gambling-related psychosocial harms. In addition, mounting losses were found to motivate the gambler to continue gambling to ‘get even’, often resulting from by irrational and erroneous cognitions [26]. However, these authors have failed to address the broad range of erroneous cognitions that maintain problem gambling behaviours, for example: (i) the permission-giving and justifying cognitions that allow the PG to create available money; (ii) minimising gambling as a problem; (iii) struggling with overwhelming emotions. These cognitions are crucial as they allow the PG to convince themselves they have money available to gamble when this is not the case [16].

The liberal provision of credit to PGs by financial institutions continues to be standard practice and does not meet the conduct expected in a banking culture, which is to act fairly, provide services that are fit for purpose, and deliver services with reasonable care and skill [27] (p. 52).

### 4.4. Recommendations

Financial Institutions should examine their duty of care to its customers, whose vulnerability, problematic credit access and credit use have been allowed to continue despite the explicit knowledge of misuse and problems. It may be that such lending practices have constituted a breach of the duty of care to these customers over a long period of time. This report focusses explicitly on one such problematic behaviour by PGs, which could potentially be recognised and responded to electronically as a public health intervention.

Swanton et al. [7] recommend that financial institutions could develop algorithms that detect potentially risky gambling transactions as described in this research. They also that suggest targeted interventions that assist financially vulnerable customers in making better gambling-related decisions for their long-term interests should be available in the general use of credit by PGs.

Research collaborations with financial institutions would be beneficial to refine consumer protection policies related to the financial products available and to develop and provide preventative interventions to improve consumer wellbeing [26]. This recommendation fits well with the findings of this research, which demonstrates that consumer protection is critical to enhancing the welfare of gamblers. As such, banks need to develop banking cultures that are responsive to the needs of their vulnerable PG customers. These could include real-time interventions by electronically blocking or deactivating cards across all accounts, which could be developed and implemented to assist PGs as part of the duty of care owed to them by the financial institution.

In a recent, Canadian court case, it was suggested that Casinos could be liable for deliberately ignoring or exacerbating their patrons’ accumulating losses [28]. Similarly, banks also have a duty of care to PGs, as they too have specific knowledge to identify common indicators of financial difficulty [29]. Recently, the Australian banks have re-written their Code of Practice to provide better protection for customers [29]. This code is timely and provides an opportunity for the credit industry to work closely with the gambling help industry to identify PG credit use gambling-related problems and support pathways, the attendance of which could be required to regain access to e-credit.

Caution is needed with regards to any positive impacts of these Codes of Practice. For example, Davis [30] argues that the Hayne Royal Commission into the Australian financial sectors findings into misbehaviour will not provide a lasting solution as the fundamental issues driving this misconduct have not been examined, so any benefits would be short-lived and the misconducts would be “likely to resurface, albeit in different guises” (p. 200). This point highlights the importance of systematically addressing irresponsible banking practices. For example, when the customer’s banking statements display a frantic use of credit and money use indicative of problematic gambling behaviours, it is essential to highlight the importance of identifying irresponsible gambling practices to protect the PG from the risk of bankruptcy [31]. Future research is needed to explore which PGs are the most vulnerable to declaring bankruptcy and when an intervention would be most useful in stopping the progression to bankruptcy [32] resulting from the financial devastation they experience by repeatedly accessing credit.

Another approach to identifying gambling-related debt problems is by healthcare providers in the context of a medical or mental health consultation by adding screening questions about financial losses and gambling spending as part of routine intake assessments [33]_._ Credit counsellors also play a critical role in improving the economic future of their clients and families and the overall quality of life of the gambler [33], for example, by providing an effective intervention that will help reduce the relapse cycle described in Figure 1, where PGs cannot think critically about their financial decisions. Financial counsellors must understand the significance of these facilitatory and permission-giving cognitions in the relapse process so they can assist the PG to address these thinking patterns to help reduce the likelihood of them entering the zone. This approach will help to break the relapse cycle by reducing the urge that is fueled by the gambler’s facilitatory and permission-giving cognitions [16].

The findings also highlight the potential need for interventions that not only address and support better financial decision-making directly, but also recognise that there are likely many other factors, such as supportive relationships that can either cause social dysfunction or offer support to help mitigate the processes described in this paper. One example is a greater focus on research to understand the role of the family of gamblers who do not seek help for their gambling problems [34].

Education about this cycle and the associated cognitions and financial risks could also assist PGs and their significant others to reduce shame and guilt in the former and to facilitate acceptance and unconditional positive regard [35] in the latter, facilitating realisation and recovery as well as early help-seeking and engagement in appropriate CBT programs [34]. This qualitative study is limited in its generalisability because of the small number of subjects. Small numbers were similarly described as a limitation by Schüll [14]. Nevertheless, because such studies are currently sparse, these are important pilot data that require serious consideration and further verification by financial institutions in line with their duty of care to their PG customers; and by researchers and treatment providers seeking to intervene earlier and more effectively. The role and duty of care of gambling venues specific to the use of credit also required further investigation and consideration. Whether this phenomenon relates to machine design (hardware and software) or not, as hypothesised by Schüll [14], there remains an uncertain but crucial question of liability. For example, a significant threat to public health approaches is the vested interest of those who can oppose or undermine policies that might reduce profits [36]_,_ such as financial institutions.

## 5. Conclusions

These findings have significant implications for prevention and harm minimisation about problem gambling. There are likely many interventions at all levels (from policy and regulatory changes, through to interpersonal support and therapy) that could be understood better, to help address this problem. As a first important step, if financial institutions identified customers demonstrating frantic use of money and credit within gaming venues and encouraged these customers to seek help, such harm could be averted, and much earlier presentation for counselling and treatment for EGM PGs could follow.

## Figures and Tables

**Table 1:** Focus group participants.

Focus Groups	Title
Participants	n = 10
Clients who completed CBT	5
Pokies Anonymous members	5

**Table 2:** In-depth interview participants.

In-Depth Interviews	Title
Participants	n = 19
Treatment Seekers Total	7
Female	2
Male	5
Pokies Anonymous Total	6
Female	4
Male	2
Non treatment Seekers Total	6
All female	6
